# Advances in Multiple Sclerosis Research–Series I

**DOI:** 10.3390/brainsci10110795

**Published:** 2020-10-29

**Authors:** Vasso Apostolopoulos, John Matsoukas

**Affiliations:** 1 Institute for Health and Sport, Victoria University, Melbourne 8001, Australia; imats1953@gmail.com; 2 NewDrug, Patras Science Park, 26500 Patras, Greece; 3 Department of Physiology and Pharmacology, Cumming School of Medicine, University of Calgary, Calgary, AB T2N 4N1, Canada

**Keywords:** multiple sclerosis, MS, vaccine, immunomodulation, carriers, MS drugs

## Abstract

Designing immunotherapeutics, drugs, and anti-inflammatory reagents has been at the forefront of autoimmune research, in particular, multiple sclerosis, for over 20 years. Delivery methods that are used to modulate effective and long-lasting immune responses have been the major focus. This Special Issue, “Advances in Multiple Sclerosis Research—Series I”, focused on delivery methods used for immunotherapeutic approaches, drug design, anti-inflammatories, identification of markers, methods for detection and monitoring MS and treatment modalities. The issue gained much attention with 20 publications, and, as a result, we launched Series II with the deadline for submission being 30 April 2021.

## 1. Multiple Sclerosis

The World Health Organization estimates that globally, more than 2.5 million people are affected by multiple sclerosis (MS). With the global population growing to an unparalleled height of 7.0 billion in 2011 and recently reaching 7.8 billion (10 October 2020)—it is estimated to reach 8.5 billion by 2030 and 9.7 billion by 2050—the incidence and onset of MS in young adults is expected to rise exponentially, with an estimate of 2.3 million people living with MS globally. Clinical isolated syndrome is a type of MS which may or may not progress. As such, a person will experience a neurological episode lasting at least 24 h and resulting in damage to the central nervous system (CNS). There are three main subtypes of MS, (i) relapse/remitting MS (RRMS) accounting for 85% of MS cases, with 50% progressing to (ii) secondary progressive MS (SPMS), with (iii) 15% of those diagnosed at onset of primary progressive MS (PPMS) type. It is possible that RRMS patients can remain in that state for up to 30 years, whilst 8% develop a more aggressive disease, named highly active RRMS (HARRMS). In rare occasions, up to 5% are progressive relapsing MS type (PRMS), which is characterized by progressive worsening of the condition from the onset, similar to PPMS.

**Figure 1:**
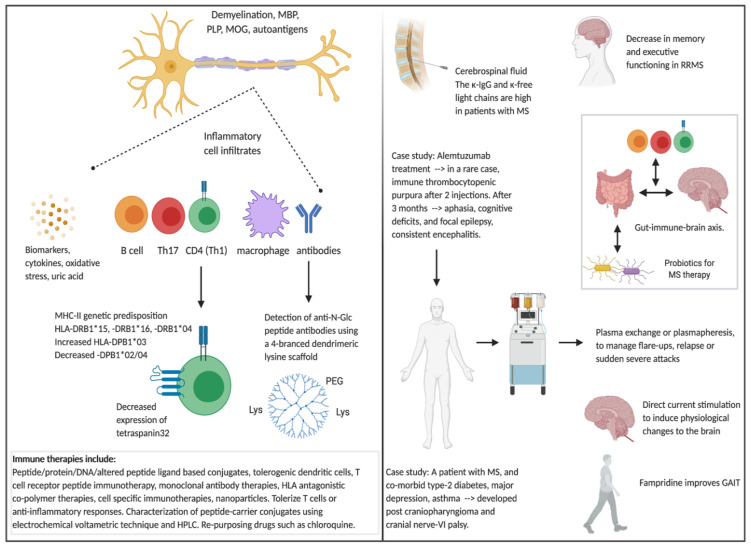
Summary of new advances in Multiple Sclerosis Research—Series I, papers in the Special Issue. Created with biorender.com.

MS is characterized as a chronic demyelinating disorder of the CNS with inflammatory cells infiltrating around the nerve, leading to demyelination of the myelin sheath and immune attack to myelin basic protein (MBP), proteolipid protein (PLP) and myelin oligodendrocyte glycoprotein (MOG). Inflammatory cells which have been found to be involved in MS include macrophages, T helper type 1 (Th1) cells, Th17 cells, CD8+ T cells and B cells secreting auto-antibodies [1,2,3]. More recently, it has been shown that tetraspanin-32 is significantly downregulated in Th cells. Tetraspanin-32 controls the development of autoimmune responses, and in EAE models in mice, tetraspanin-32 is significantly expressed at lower levels on activated or encephalitogenic T cells compared to naïve Th cells. In the study by Basile and Cavalli et al., it was noted that tetraspanin-32 was downregulated in memory T cells and was further decreased upon ex vivo restimulation (Figure 1) [4]. Likewise, myelin-specific memory T cells and peripheral blood mononuclear cells (PBMC) from patients with MS also expressed lower levels of tetraspanin-32 compared to memory T cells from healthy subjects. In addition, MS patients with early relapses compared to those with a longer, stable disease expressed lower levels of tetraspanin-32 on their PBMC [4]. Hence, tetraspanin-32 is involved in immune responses underlying the pathophysiology of MS, and could be a viable diagnostic marker or therapeutic target against MS.

A number of factors contribute to MS development, including genetic predisposition, especially those who are HLA-DR2 (HLA-DRB1*15, HLA-DRB1*16)- or HLA-DR4 (HLA-DRB1*04)-positive, environmental factors such as Epstein–Barr virus and human herpesvirus 6 exposure, and diet, such as low levels of vitamins B and D [1,5,6]. A number of health conditions are related to HLA phenotype, such as type-1 diabetes (HLA-DRB1*03 or HLA-DR3, HLA-DQB1*03 or HLA-DQ8), rheumatoid arthritis (HLA-DRB1*04), juvenile idiopathic arthritis (HLA-DRB1*08), celiac disease (HLA-DQ2, HLA-DQ8) and Graves’ disease (HLA-DRB1*03, HLA-DQA1*0501). The paper by Maria Anagnostouli et al. studied the prevalence of HLA-DPB1 allele in MS patients from a Greek cohort and its association with HLA-DRB1 risk allele [7]. No significant differences were noted between early onset MS compared to adult onset MS for 23 distinct HLA-DPB1 and 12 HLA-DRB1 alleles. However, the frequency of HLA-DPB1*03 allele was significantly increased, and the frequency of HLA-DPB1*02 allele was significantly decreased, in AOMS patients compared to controls. Interestingly, the frequency of HLA-DPB1*04 allele was significantly decreased in both patients, with early onset and adult onset MS compared to controls, suggesting a protective role of this allele amongst Greek cohort patients (Figure 1) [7]. Koukoulitsa and colleagues present a nice review articulating the journey of the conformational complex between HLA-peptide with the T cell receptor of agonist peptides and their altered peptide ligands from MBP, MOG and PLP [8].

## 2. Detection and Monitoring of Patients with MS

Magnetic resonance imaging (MRI) has been the gold standard of diagnosing and monitoring disease by detecting brain lesions and the type of brain lesion which aids treatment decisions. In addition, other detection methods are used in combination with MRI, such as the Kurtzke Expanded Disability Status Scale (EDSS) which measures the body’s function and how well it can move, as well as analysis of cerebrospinal fluid for free light chains and IgG. Together, these increase the accuracy of diagnosis of MS and are used to monitor disease progression. However, there are few simple assays available to follow up disease activity. As such, the detection of auto-antibodies from sera is a method to detect relevant biomarkers. The team by Nuti and Papini et al., developed a method to detect anti-N-glycosylated (N-Glc) peptide antibodies, using a four-branched dendrimeric lysine scaffold, linked to a polyethylene glycol-based spacer containing 19-amino acids. This efficient multivalent probe has specificity and high affinity for anti-N-Glc antibodies in patients with MS [9]. In addition, Gudowska-Sawczuk evaluated cerebrospinal fluid and sera from patients with either MS (*n* = 34) or other neurological disorders (*n* = 42) [10]. The concentrations of cerebrospinal fluid κ free light chains (κFLC) and λFLC, and sera κFLC, as well as κFLC, λFLC, and κIgG index, were significantly higher in patients with MS compared to those with other neurological disorders. The κIgG index showed the highest diagnostic power in the detection of MS with both κFLC index and κIgG indexes showing the highest diagnostic sensitivity. This study provides novel information about the diagnostic significance of four markers combined in the κIgG index [10] and shows that κFLC and κIgG combined in a novel algorithm may improve the detection and disease activity of MS (Figure 1).

Cognitive function refers to a range of high-level brain functions, such as the ability to learn and remember information, solve problems, focus, concentration, attention, and verbal fluency. Change in cognitive function is common in patients with advanced MS. However, Pitteri et al, showed that newly diagnosed RRMS patients (*n* = 50) performed worse than healthy controls (*n* = 36), in particular, in the domains of memory and executive functioning [11]. These data demonstrate that reduced cognitive functioning can be present early on during the course of disease, even in patients without evidence of cognitive impairment. As such, the cognitive impairment criteria for patients with MS should be re-evaluated and be monitored closely throughout the course of disease (Figure 1).

## 3. Treatments for MS

Treatments for MS include, interferon (IFN) beta-1a, IFN beta-1b (cytokines), fingolimod, ozanimod, siponimod (sphingosine-1-phosphate-receptor modulators), natalizumab (a monoclonal antibody against alpha4-integrin), dimethyl fumarate, glatiramer acetate, teriflunomide, cladribine, ocrelizumab (a humanized anti-CD20 monoclonal antibody) and, alemtuzumab (a humanized anti-CD52 monoclonal antibody) [1,2]. These drugs are focused on speeding recovery from relapse, slowing the progression of disease and managing MS symptoms, and in most cases, there are side effects and patients need to stop treatment due to non-tolerance of the treatment. In rare cases, more severe adverse events occur. In fact, Buscarinu et al., presented a case report of a 45 year old Italian woman with RRMS on alemtuzumab treatment who showed immune thrombocytopenic purpura after the second injection of alemtuzumab. Three months following treatment, the patient presented with transient aphasia, cognitive deficits, and focal epilepsy, consistent encephalitis [12]. Autoimmune complications following alemtuzumab treatment are generally rare, with only one previous case being reported. Furthermore, Sachinvala et al. reported a male patient with MS, and co-morbid type-2 diabetes, major depression, asthma, developed post craniopharyngioma and cranial nerve-VI palsy. Magnetic resonance imaging, Humphrey’s visual filed and retinal nerve fiber thickening were used to determine changes to help the patient maintain productivity and mental state and mood (Figure 1) [13].

There is a need for the development of new treatment options which would stop progression and have little to no side effects. Immune therapies have come a long way in recent years, with a number of methods being tested in pre-clinical and clinical settings, such as peptide/protein/DNA based vaccines, tolerogenic dendritic cells, T cell receptor peptide immunotherapy, monoclonal antibody therapies (anti-integrin a-4, anti-leucine rich repeat and immunoglobin-like domain-containing protein 1 (LINGO-1), anti-CD52), HLA antagonistic co-polymer therapies, cell specific immunotherapies, peptide-carrier conjugates, all of which are extensively reviewed by Kammona and Kiparissides [14] and Metaxakis et al. [15] (Figure 1). An editorial entitled, the long road of immunotherapeutics against MS [16], highlighted 20 years of MS research of an international multi-disciplinary consortia including peptide chemistry, medicinal chemistry, protein synthesis, protein–peptide interactions, nuclear magnetic imaging, molecular modeling, molecular dynamics, molecular biology, immunology, cell biochemistry, animal research and clinical research. This multi-disciplinary consortia led to at least 10 immunotherapeutic peptide-carrier candidates to be tested in human clinical trials. In preclinical studies, these peptide-based immune modulating conjugates showed a safety profile whilst switching immune responses from pro-inflammatory to anti-inflammatory and protection against experimental autoimmune encephalomyelitis (EAE) in mouse models [3,17,18,19,20,21,22,23,24]. Characterization of peptide-carrier conjugates was demonstrated using electrochemical voltametric techniques and high-pressure liquid chromatography [25]. In addition, nanoparticles have been used to deliver MS antigens to the immune system to tolerize T cells or stimulate an anti-inflammatory responses, reviewed by Chountoulesi and Demetzos [26]. More recently, chloroquine, an anti-malarial drug, was shown to suppress EAE in mice by modulating dendritic cells, Th17 cells, astrocytes, oligodendrocytes and microglia. Microglia cells were also shown to secrete IL-10 and IL-12p70. These data provide evidence that drug repurposing of chloroquine may be useful to patients with MS (Figure 1) [27].

In the last ten years, the incidence of MS has increased considerably, with lifestyle and environmental factors being one of the main contributors. An informative review by Boziki and Grigoriadis et al., provide the current advances in the gut-microbiome-immune–brain axis in patients with MS with altered microbiome, and present the effects of MS treatments on gut microbiome (Figure 1) [28]. Thus, modification of gut microbiota by either dietary (such as, probiotics) or medicinal approaches is a promising approach for the management of MS. In fact, probiotics have been shown to have beneficial effects not only in the gut flora but also in modulating and maintaining a healthy immune system. Certain probiotics have been shown to have anti-inflammatory effects on immune cells (i.e., monocytes) and in disease settings, such as asthma and allergies [29,30]. The paper by Dargahi et al. showed that the probiotic *Streptococcus thermophilus* was able alter pro-inflammatory T cells responses against an agonist MBP_83–99_ peptide to an anti-inflammatory profile (Figure 1) [31]. This study suggests that the consumption of *Streptococcus thermophilus* may be beneficial in the management and treatment of autoimmune diseases such as MS, and further research in this area is warranted.

In addition to intravenous or oral steroids that are used as the first line of therapy for MS relapse, therapeutic plasma exchange, or plasmapheresis, is another method used to treat patients with neuromyelitis optica spectrum disorders, autoimmune encephalitis and MS, especially those with sudden, severe attacks or relapse/flare-ups. It is used in MS patients to manage disease by exchanging their plasma with ‘fresh’ plasma to remove pro-inflammatory cytokines and other proteins involved in auto-immune attack. In a study published by Moser et al., in this Special Issue, they compare the indications, efficacy and safety of therapeutic plasma exchange treatment in MS, autoimmune encephalitis and other immune-mediated CNS disorders and noted consistent efficacy and safety [32]. Measuring biomarkers of inflammation and oxidative stress is important to understand the efficacy of treatments. As such, Moccia et al. studied 60 patients with RRMS who were treated with IFN beta-1a or Coenzyme Q10 and monitored patients for IL-1b, IL-2R, IL-3, IL-4, IL-5, IL-6, IL-7, IL-8, IL-13, RANTES, tumor necrosis factor and uric acid (Figure 1) [33]. These serum biomarkers could be used to determine the efficacy of treatments as well as their mechanisms of action.

It is believed that transcranial magnetic stimulation motors with direct current stimulation (tDCS) intensities induce physiological changes to the brain, although the mechanism of action, as well as its validity and efficacy, are not clear. In a pilot study by Workman and colleagues, they noted that there were no immediate changes in cerebral blood flow following direct current stimulation. Hence, further work is required to enable sufficient magnitudes of intracranial electrical fields to induce physiological changes in the brain to patients with MS (Figure 1) [34]. During disease progression, patients with MS develop walking limitations, and fampridine is usually recommended to improve gait. In the study by Ahdab et al., fampridine was evaluated for cortical excitability effects and whether changes could predict therapeutic responses in 20 patients with MS and gait impairment [35]. It was noted that fampridine increased the excitatory intracortical processes, as shown by paired-pulse transcranial magnetic stimulation, suggesting that this could be used to select patients with MS who would be likely to experience a favorable response to fampridine (Figure 1) [35].

## 4. Conclusions

The development of drugs, immunotherapeutics and vaccines against diseases is a long process often taking researchers a lifetime. In this Special Issue, “Advances in Multiple Sclerosis Research—Series I”, a range of papers were published, including MS markers, treatments, detection, monitoring and the role of the microbiome in MS. Together, all this information advances our knowledge of MS research, with promising new leads being developed in the next few years and entering human clinical trials.
